# Uropathogenic *Escherichia coli* causes significant urothelial damage in an *ex vivo* porcine bladder model, with no protective effect observed from cranberry or d-mannose

**DOI:** 10.1093/femspd/ftae026

**Published:** 2024-10-03

**Authors:** Jenane Konesan, Kate H Moore, Kylie J Mansfield, Lu Liu

**Affiliations:** School of Biomedical Sciences, UNSW Sydney, Sydney, NSW 2052, Australia; St George Hospital, UNSW Sydney, Kogarah, NSW 2217, Australia; Graduate School of Medicine, University of Wollongong, Wollongong, NSW 2522, Australia; School of Biomedical Sciences, UNSW Sydney, Sydney, NSW 2052, Australia

**Keywords:** urinary tract infection (UTI), uropathogenic *escherichia coli* (UPEC), urothelial integrity, cranberry, d-mannose

## Abstract

Urinary tract infections (UTIs), primarily caused by uropathogenic *Escherichia coli* (UPEC), have an unclear impact on bladder mucosal physiology. This study investigates UPEC’s effects on the urothelium and lamina propria using an *ex vivo* porcine bladder model. Bladder mucosal strips were analysed for contractile responses to acetylcholine, serotonin, and neurokinin A. Given rising antibiotic resistance, non-antibiotic agents such as cranberry and d-mannose were also evaluated for their potential to prevent UPEC-induced damage. The findings of the current study revealed that UPEC significantly compromised urothelial integrity, barrier function, and permeability, with loss of urothelial cells, uroplakins, and tight junction protein ZO-1 expression. Additionally, infected bladders exhibited a markedly enhanced contractile response to serotonin compared to uninfected controls. Notably, neither cranberry nor d-mannose offered protection against UPEC-mediated damage or mitigated the heightened serotonin-induced contractility. This study provides novel insights into how UPEC disrupts bladder cell biology and highlights the possible involvement of serotonin in the pathophysiology of UTIs.

## Teaser

This study reveals that Uropathogenic *Escherichia coli* causes significant damage to bladder mucosa, affecting cell integrity, barrier function and contractility, highlighting the potential role of serotonin in bladder infections.

## Introduction

Urinary tract infections (UTIs) are defined by the presence of bacteria within the urinary tract, most commonly within the urinary bladder (Stamm and Norrby 2001), and are commonly associated with an inflammatory response. UTIs are the most common bacterial infections in humans, affecting an estimated 150 million people worldwide each year (Stamm and Norrby 2001). More than 50% of women will experience a UTI in their lifetime, and up to 50% of them will endure recurrent infections within 6 months (Foxman 2002). UTIs result in considerable patient morbidity and a significant reduction in the patient’s quality of life (Sihra et al. 2018). Hence, the management of this condition has incurred significant financial costs, estimated at $900 million in Australia per year, and is expected to reach $1.5 billion per year by 2030 (Consortium 2020).

Bladder infections are primarily caused by the Gram-negative bacteria, uropathogenic *Escherichia coli* (UPEC) (Terlizzi et al. 2017), which initiates ~75% of UTIs in women (Medina and Castillo-Pino 2019). These organisms infect and cause damage to the urinary bladder, potentially disrupting the critical barrier functions of the urothelium (Khandelwal et al. 2009, Acharya et al. 2004). The urothelium, the lining of the bladder, usually forms a tight barrier preventing any harmful solutes and bacteria from permeating into the sub-epithelial space of the bladder (Floyd et al. 2010). UTI arises from adherence of UPEC type 1 fimbriae to uroplakin (UP) proteins on the apical surface of the urothelium (Terlizzi et al. 2017). UPEC is then internalized within the host cell (Mulvey et al. 2001, Schwartz et al. 2011, Justice et al. 2004). Intracellular growth of UPEC reduces the viability of the urothelium and thus triggers urothelial cell death (Klumpp et al. 2006). This subsequently disrupts the barrier function, causing significant loss of tight junction proteins such as ZO-1 (Tian et al. 2018).

The tissue damage caused by UPEC is associated with increased production of reactive oxygen species (ROS) (Khandelwal et al. 2009), which have antimicrobial properties. However, although this defence mechanism plays a vital role in the immune response, it can also be destructive, as excessive ROS production can cause tissue damage (Laskin et al. 2011). Many inflammatory diseases are characterized by ROS-induced tissue damage (Yang and Lian 2020). The imbalance between ROS and the body’s defence system can lead to significant oxidative stress (Freeman and Crapo 1982, Pizzino et al. 2017). The excessive tissue damage and loss of the urothelial barrier exposes the sub-urothelial afferent nerves, possibly acting as a trigger for the symptoms commonly associated with UTIs, including pain, frequency, and urgency in patients (Bono et al. 2023). Tissue damage may also enhance contractility of the bladder mucosal layer (urothelium and lamina propria) (Bono et al. 2023), which can be activated by a number of receptors including muscarinic (Moro et al. 2011), 5-hydroxytryptamine (5-HT, also known as serotonin)(21), and neurokinin receptors (Grundy et al. 2018).

UTIs are typically treated with antibiotics as the first-line therapy (Kwok et al. 2022). However, UTIs are becoming more difficult to treat due to the substantial rise in antibiotic resistance (Samanci and Pınarbaşı 2023). As a result, there is significant demand to identify non-antibiotic alternatives (Sihra et al. 2018). Several non-antibiotic agents have been examined in clinical trials, with the main agents showing some efficacy being cranberry and d-mannose. *In vitro* studies, both cranberry and d-mannose have been shown to inhibit the binding of UPEC to the urothelium but both agents have shown varying degrees of success in clinical trials for the prevention of UTIs in women (Sihra et al. 2018). In our previous cell culture study, cranberry, but not d-mannose, prevented UPEC-induced cell damage and cell death in a urinary tract cell culture model using Madin-Darby canine kidney (MDCK) cells. Hence, this compelling result provided a rationale for further exploration of whether cranberry and d-mannose could provide a protective effect against UPEC-damage in an *ex-vivo* porcine bladder.

The aim of this research was to develop an *ex-vivo* UPEC-infected porcine bladder model to assess the impact of UPEC on the urothelial integrity, viability, and barrier function. Additionally, this study investigated the level of oxidative stress when UPEC is present. This study also evaluated the ability of non-antibiotic agents, cranberry and d-mannose, to prevent UPEC-induced changes. The contractility of the mucosa was also examined in response to acetylcholine (ACh), serotonin (5-HT), and neurokinin A (NKA).

## Methods

### Porcine bladder specimen preparation

Female pig bladders were chosen as an acute model of UTI in this study, as they contains similar anatomical and structural features to the human bladder (Nielsen et al. 2019). Female pig bladders (6–9 months old) were collected from a local abattoir and transported on ice to the laboratory (~2 h). Upon arrival in the laboratory, any connective and fat tissues on the outer surface of the bladder were removed and bladders were rinsed with Krebs–Henseleit solution (composition in mM: NaCl 118, KCl 4.7, NaHCO_3_ 25, KH_2_PO_4_ 1.2, MgSO_4_ 1.2, CaCl_2_ 2.5, and d-glucose 11.7, bubbled with carbogen to stabilize the pH to 7.4), supplemented with 1% penicillin–streptomycin (PS).

### *Ex*-*vivo* model of UTI

**Figure 1.**
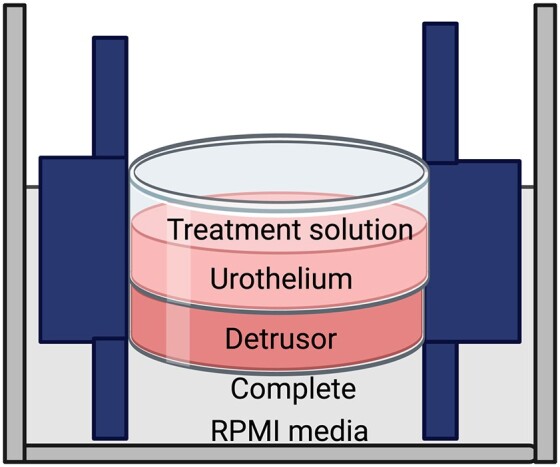
Schematic figure of the incubation chamber. Full-thickness sheets of bladder were sandwiched between two separated bathing solutions. The serosa side of the bladder sheet was placed facing the bottom chamber which contained complete RPMI media (10% FCS and 1% PS). The luminal side of the urothelium, facing the top chamber, was immersed in antibiotic-free complete RPMI media with 10% FCS along with different treatments, including control [antibiotic-free RPMI (AF-RPMI)], cranberry, d-mannose, UTI89, cranberry with UTI89 or d-mannose with UTI89. The tissues were incubated at 5% CO_2_/95% O_2_ for 4 h at 37°C (Moro and Edwards 2016) before isolation for staining and pharmacological analysis.

After rinsing the bladders, each bladder was cut open from the urethra to the dome. Segments of bladder dome (size 50 mm × 50 mm) were placed in chambers (Smith et al. 2014) with media separately bathing the luminal and serosal surfaces (Wellens et al. 2008) (Fig. 1). The luminal surface (urothelium) was treated with the control, antibiotic-free Roswell Park Memorial Institute (AF-RPMI) media, cranberry (3 mg/ml) or d-mannose (10 mM), with or without UPEC (UTI89, 2.0 × 10^8^ CFU/ml) for 4 h in a tissue culture incubator at 37°C, 95% CO_2_. The serosal surface was maintained in complete RPMI media containing 10% FCS and 1% PS.

### Bacterial growth and pre-treatments

The UPEC species used for these studies was UTI89, obtained from Prof. Mark Schembri, University of Queensland. This isolate was first cultured from urine collected from a young female patient with an acute UTI. UTI89 has been used for many previous laboratory studies to characterize the interaction of UPEC with the urothelium (Mysorekar and Hultgren 2006, Conover et al. 2016, Bokil et al. 2011). A single bacterial colony of UTI89 was resuspended in 10 ml of LB broth growth medium and grown overnight at 37°C. On the day of treatment, 1 ml of bacteria was resuspended in 9 ml of LB broth and incubated at 37°C for another 1 h.

In the meantime, cranberry powder (Eclectic Institute 30 138) was dissolved in AF-RPMI, supplemented with 10% FBS to a final concentration of 3 mg/ml. The mixture was vortexed for 30 s and sterilized by filtration. d-mannose powder (Sigma M8574) was dissolved in AF- RPMI to a final concentration of 10 mM and sterilized through filtration. It is worth noting that the optimal cranberry (1, 3, 10, and 30 mg/ml) and d-mannose (1, 10, 30, and 100 mM) concentrations were determined in a previous cell culture study using MDCK cells (Konesan et al. 2023). The exact concentrations of cranberry and d-mannose that can reach the urinary bladder have not been established yet. However, the concentrations used in our study align with previously published studies. For instance, a concentration of 3 mg/ml was chosen for cranberry due to its alignment with the linear curve in Gupta et al.’s investigation (Gupta et al. 2007). The chosen concentration for d-mannose (10 mM) was consistent with Wellen. et al.’s findings (Wellens et al. 2008). In our previous cell culture study, d-mannose exceeding 30 mM demonstrated a toxic effect on the cell monolayer (Konesan et al. 2023). Additionally, preincubation of MDCK cells with cranberry and d-mannose was evaluated prior to the addition of UTI89. Hence, these concentrations were used to determine if a similar outcome would be observed in this *ex-vivo* porcine bladder model.

Following the 1-h incubation, the bacteria were diluted to an optical density (OD600) of 0.4 (measured using Spectronic 20D+, Thermo Fisher Scientific), equivalent to 2.0 × 10^8^ colony forming units. UTI89 was centrifuged at 5000 rcf and resuspended in equal amounts of AF-RPMI), or AF-RPMI containing cranberry or d-mannose for 90 min at room temperature before being applied to the luminal surface of the porcine bladder and incubated for 4 h, as mentioned above.

### Immunohistochemistry staining of porcine bladder tissue

#### Processing of tissue

Intact sections of each bladder wall (1 cm × 1 cm) were collected from the dome of the bladder and fixed in Zamboni’s fixative (containing paraformaldehyde 2%, NaH_2_PO_4_ 0.3%, Na_2_HPO_4_ 3%, picric acid 0.4%, water). Fixed intact segments were processed and embedded into paraffin. Sections (5 µm) were cut and mounted onto poly-l-lysine coated slides.

### 3,3′-Diaminobenzidine staining and immunofluorescence

For 3,3′-diaminobenzidine (DAB) staining and immunofluorescence, mounted sections were dewaxed in xylene prior to incubation in the antigen retrieval chamber. Following antigen retrieval with 3% universal retrieval solution (Abcam ab208572), slides were incubated for ~15 min at room temperature with 3% hydrogen peroxide. The slides were washed three times with 1 × phosphate-buffered saline (PBS) and incubated in 10% donkey blocking serum (Sigma 09 663) diluted in PBS for 1 h at room temperature.

For DAB staining, the primary and secondary antibodies were diluted in Tris-buffered saline with 0.05% Triton (TBS-TX) and 2% donkey or goat serum to the specified ratios as outlined in Supplementary Table S1. The negative controls were incubated in TBS-TX buffer only. The slides were incubated overnight at room temperature with primary antibodies. For immunofluorescence, the primary antibody was added with 1% blocking serum in TBS-TX and was incubated overnight and wrapped in foil.

On the subsequent day, for both methods, the slides were washed for 3 × 10 min with TBS buffer and incubated with secondary antibody for 2 h at room temperature (Supplementary Table S1). The slides were washed again with TBS for 3 × 10 min.

For DAB staining, DAB signal development slides were incubated for 45 min with preincubated Avidin-Biotin Complex solution, slides were washed with TBS for 3 × 10 min, and DAB solution was added to develop signal for 3 min. The slides were counterstained with hematoxylin and Scott’s blue solution and coverslips applied. For immunofluorescence, the sections were washed for 3 × 10 min in TBS. DAPI (30 µl) mounting medium was then placed over the tissue to stain the nuclei blue.

### Visualization and analysis of stained tissue slides

Both DAB immunohistochemistry and immunofluorescence slides were scanned at 20× magnification using the Neurolucida microscope system. The positive staining of uroplakin III immunoreactivity (UP-IR), 8-oxo-dG-IR, and caspase-3-IR under different treatment conditions were quantitatively analysed with QuPath. DAB staining for UP-IR, caspase-3-IR, and 8-oxo-2′-deoxyguanosine-IR (8-oxo-dG-IR) were quantified by setting the staining in the muscle layer as the base threshold (background). Values exceeding this were considered positive UP-IR, caspase-IR, or 8-oxo-dG-IR expression. For UP-IR, areas (50 000 µm^2^) were selected throughout the entire urothelium to calculate the DAB staining %. For both 8-oxo-dG-IR and caspase-3-IR, consistent areas were chosen through the urothelium (50 000 µm^2^) and lamina propria (250 000 µm^2^). For each stained tissue slide, data were calculated for 20 to 30 areas per slide, 5 slides per animal, and 5 animals per treatment.

Tissue damage was scored based on the criteria listed in Table 1 to assess the urothelial (mean score out of 3) and submucosal injury (mean score out of 4), one mean score was applied to each field of view (Erben et al. 2014). Five to eight slides of each treatment and control samples were evaluated, and for each slide, ~20 to 30 fields of view were examined. The scores from each field of view were added to obtain a final mean score for the whole slide. The results were analysed using the one-way ANOVA to compare the severity of the microscopic damage between samples.

**Table 1.** Microscopic scoring method for bladder mucosal damage.

Type of Injury	Severity of injury	Morphology	Score
Urothelial	No damage	Urothelium intact	0
	Mild	2/4 cell layers intact	1
	Moderate	1/4 cell layers intact	2
	Severe	No urothelium left	3
Oedema	Absent	0	0
	Mild	25%	1
	Moderate	50%	2
	Severe	75%	3
	Very severe	75%–100%	4

### Contractility studies of porcine mucosal tissue strips

Following the perfusion, mucosal strips (5 mm × 10 mm) from each treatment (control, cranberry (3 mg/ml), d-mannose (10 mM), UTI89 (OD: 0.4), cranberry with UTI89 and d-mannose with UTI89) were obtained for organ bath experiments to investigate the contractile response of the bladder mucosa to ACh, 5-HT, and NKA.

Mucosal strips were mounted on isometric force transducers under 1 g tension in 3.5 ml organ baths containing carbogenated Krebs–Henseleit solution at 37°C and equilibrated for 60 min. Contractility was recorded as gram tension using the Polygraph computer program. An initial maximum contractile response to ACh (10^−2^ M) was generated for each strip, followed by washes and a further 30-min equilibration period. The contractility of mucosal strips in response to increasing concentrations of ACh (10^−7^ to 10^−2^), 5-HT (10^−9^ to 10^−4^), or NKA (10^−7^ to 3 × 10^−5^) was measured. Once a contractile plateau was reached for each concentration, the strips were washed, and a 30- to 60-min waiting period was allowed between each contractile response. After completing the concentration-response curves for each drug, ACh (10^−2^ M) was applied again to each strip. The average of the initial and final maximum responses to ACh for individual strips was taken as the maximum response for that strip. This was based on the observation that maximum responses to ACh remained consistent under different pre-treatment conditions (Supplementary Fig. S1).

The E_max_ was calculated via GraphPad Prism (version 10.2.0) by using the row statistics function to determine the mean and standard error mean (SEM). EC_50_ was calculated through GraphPad Prism by selecting the sigmoidal dose-response under nonlinear regression model and constraining the bottom value to ‘0’ and setting the top value to the E_max_ for each treatment.

### Statistical analysis

All data were analysed using GraphPad Prism (version 10.2.0) and presented as the mean ± SEM of 6 or more standalone experiments. Multiple group comparisons and grouped data for the severity of mucosal damage, UP-IR, caspase-IR, and 8-oxo-dG-IR data were subjected to one-way ANOVA, followed by Bonferroni’s multiple comparison test. The organ bath study used a two-way ANOVA test, followed by Bonferroni’s test. Statistical significance was determined by a *P*-value of < 0.05.

## Results

### Effect of UPEC on porcine urothelial integrity and viability

**Figure 2.**
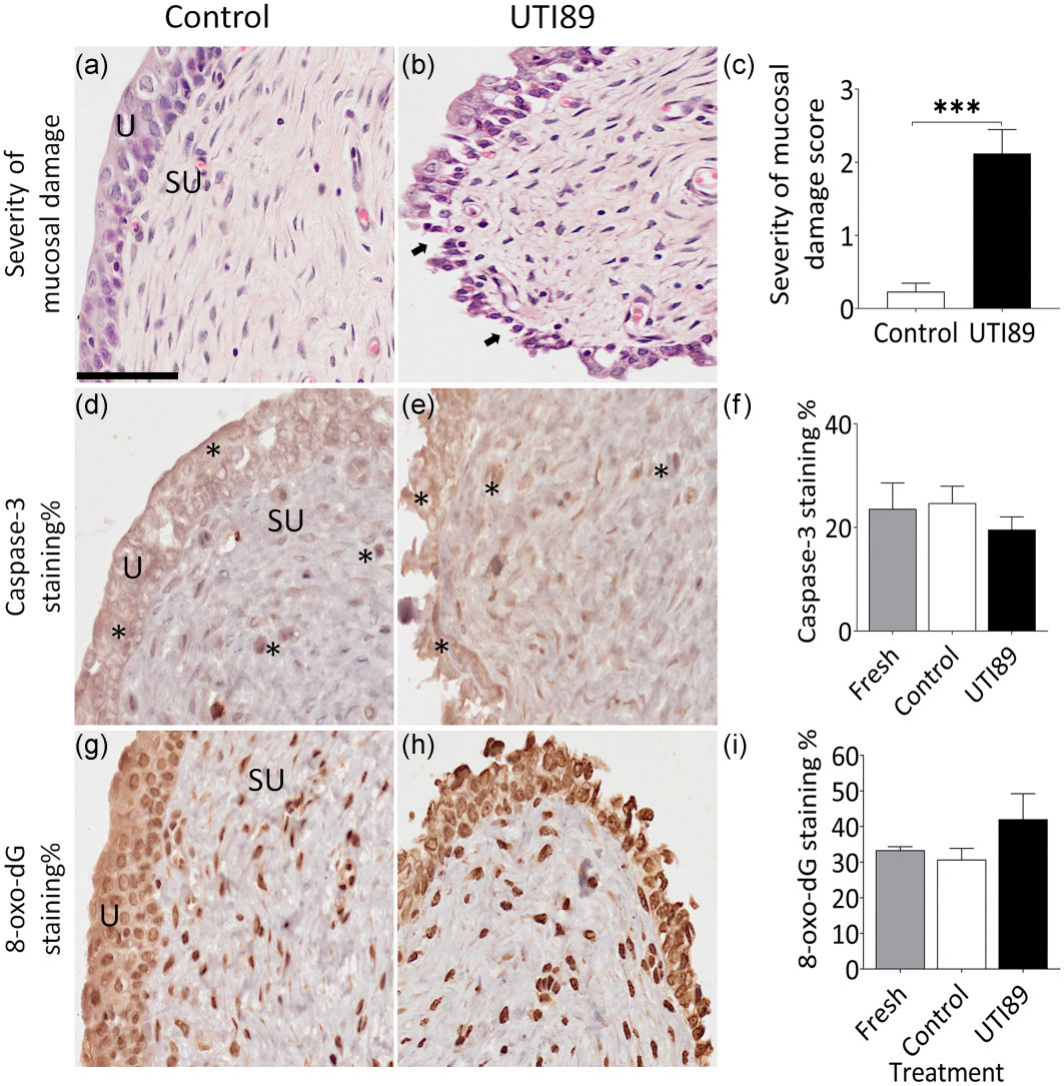
Severity of mucosal damage induced by UPEC UTI89. (a–c) H&E staining was used to visualize the mucosal layer after a 4-h treatment with control (antibiotic-free RPMI) (a) or with UTI89 (2.0 × 108 CFU/ml) (b). Panel (c) shows the severity scores of mucosal damage in comparison between control and UTI89-treated groups. The data were collected from *n* = 6 individual experiments (∼120 fields of view). ***P < 0.001, UTI89 compared to the control (one-way ANOVA, followed by Bonferroni’s comparisons test). ‘U’ denotes the urothelium and ‘SU’ denotes the suburothelium. Arrows indicate the loss of urothelial cells. The scale bar represents 100 µm. (d-f) Caspase-3-IR staining in bladder treated with antibiotic-free RPMI (control, d) or with UTI89 (e). Asterisk symbols (*) denote caspase-3-IR staining. Quantitative immunohistochemistry analysis demonstrated similar caspase-3-IR expression levels across control, UTI89 treated tissues, and freshly collected non-treated bladder specimens with no statistical difference (f). The data were collected from *n* = 5 individual experiments. (g-i) 8-oxo-dG-IR staining in bladder treated with antibiotic-free RPMI (control, g) or with UTI89 (h). Quantitative immunohistochemistry analysis demonstrated similar 8-oxo*-*dG-IR expression levels across control, UTI89 treated tissues, and freshly collected non-treated bladder specimens with no statistical difference (i). The data were collected from *n* = 5 individual experiments.

Urothelial and submucosal injury was assessed according to the criteria described in Table 1. The urothelium was well preserved in control bladder tissues (incubated with AF-RPMI, Fig. 2a). However, significant disruption to the mucosal layer was observed in tissues treated with UTI89 (Fig. 2b). This corresponded to high scores of mucosal damages (Fig. 2c), which were significantly increased compared to control (*P* = 0.0003). None to very minimal oedema was noted in the lamina propria and was therefore not reported in this paper. Caspase-3-IR was used as a measure of cell death by apoptosis within the urothelium and suburothelial layer. Caspase-3-IR staining was visible in the urothelium and submucosa of the non-treated fresh bladders. There was no difference in the caspase-3-IR expression between the control (Fig. 2d) and UTI89-infected bladders (Figs. 2e, f). The production of 8*-*oxo*-*dG-IR was used as an indicator of ROS production and hence oxidative stress in the bladder mucosal tissue. Neither the control (Fig. 2g) nor the UTI89-treated porcine bladders (Fig. 2h) demonstrated any difference in 8-oxo-dG-IR expression compared to the fresh tissue (Fig. 2i).

### Effect of UPEC-induced disruption to urothelial barrier function

**Figure 3.**
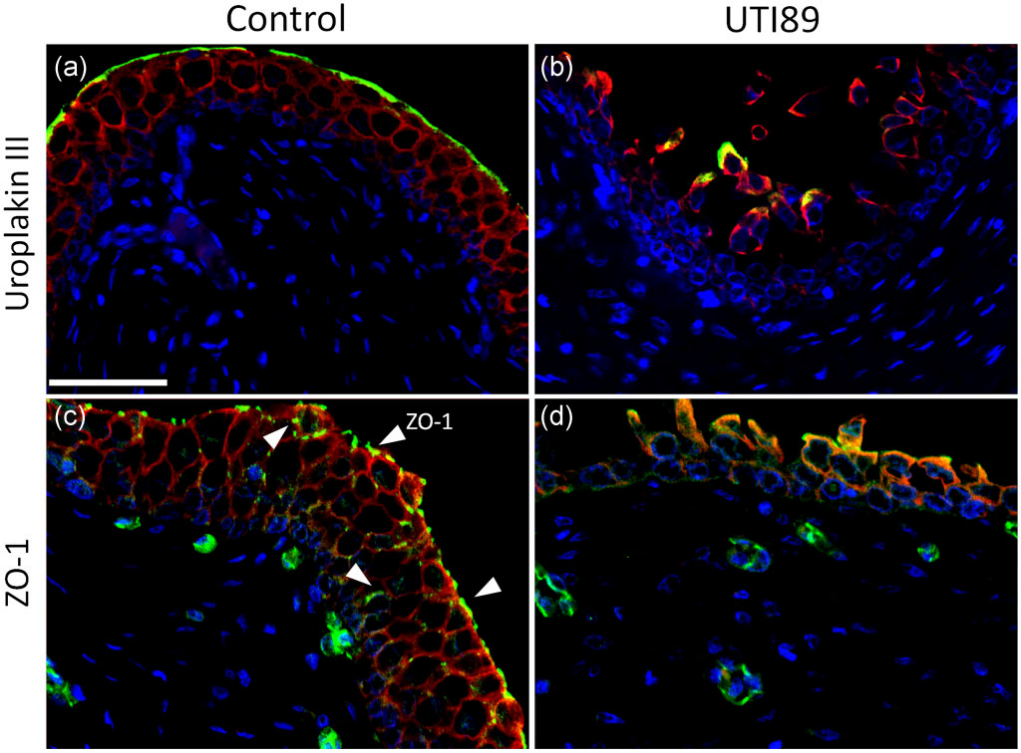
Examination of the urothelial barrier layer. Double labelling of Immunofluorescent staining of AE1/AE3, the epithelial marker (red), and uroplakin III (UP, green) in urothelium and suburothelium of bladder tissues treated with antibiotic-free RPMI (control, a) or UTI89 (b). Double labelling of Immunofluorescent staining of AE1/AE3 (red) and ZO-1 (green) of urothelium and suburothelium of bladder tissues treated with antibiotic-free RPMI (control, c) or UTI89 (d). Blue staining represents nuclei. The control demonstrated an intact urothelium morphology, expressed as a continuous layer of UP-IR (a) along the apical surface of the urothelium and a well-preserved ZO-1-IR (c), visible as small dots between the individual urothelial cell membranes. However, following incubation with UTI89 (2.0 × 108 CFU/ml), there was significant disruption and loss of the UP layer (b), and significant disturbance and loss of the ZO-1 tight junction network in the urothelium (d). The scale bar represents 100 µm.

In control porcine bladders, the UP layer was preserved on the apical surface of the urothelium (Fig. 3a). However, when porcine bladders were treated with UTI89, there was significant disruption to the UP layer (Fig. 3b). In these tissues, UP-IR expression was quantified using DAB staining (images not shown). Treatment of porcine bladder with UTI89 led to a significant (30%) reduction in UP-IR staining (expressed as UP staining %, control 18.2 ± 3.07; UTI89 treated 7.09 ± 1.91, *P* = 0.036, Table 2). In addition to the disruption to the UP layer treatment of porcine bladder tissue with UTI89 also resulted in loss of tight-junction (ZO-1) proteins. In control tissue, labelling of ZO-1 showed the expression of the tight junction protein ZO-1 as green dots located between individual urothelial cells (Fig. 3c). However, in UPEC-treated bladders, the ZO-1 expression was significantly decreased and was mostly absent in the urothelium (Fig. 3d).

**Table 2.** Comparison of different treatment groups for mucosal damage levels, caspase-3-IR, 8-oxo-dG-IR, and UPIII-IR.

Group	Control	Cranberry	d-mannose	UTI89	Cranberry + UTI89	D-mannose + UTI89
Mucosal damage score	0.23 ± 0.12	0.27 ± 0.13	0.38 ± 0.22	2.12 ± 0.33***	2.28 ± 0.22	1.87 ± 0.14
Caspase-3-IR staining%	24.7 ± 3.25	27.8 ± 3.23	34.3 ± 4.00	19.6 ± 2.45	24.6 ± 4.57	29.8 ± 5.03
8-oxo-dG-IR staining%	41.8 ± 6.45	43.9 ± 4.96	27.6 ± 3.16	39.9 ± 7.04	33.3 ± 4.42	33.9 ± 2.25
UP-IR staining %	18.2 ± 3.07	15.7 ± 4.44	14.4 ± 2.86	7.09 ± 1.91*	6.97 ± 1.92	7.32 ± 1.42

Data are shown as mean ± SEM from *n* = 5–8 bladders for each group (two-way ANOVA followed by Bonferroni test). ***P < 0.001, UTI89 compared to control for H&E mucosal damage score and *P < 0.05 for UTI89 compared to control for UP-IR analysis.

### Effect of UPEC on contractile responses of bladder mucosal strips to ACh, 5-HT and NKA

**Figure 4.**
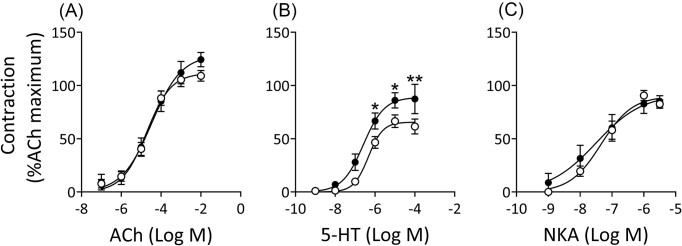
Contractile response of porcine bladder mucosal strips to ACh (a), 5-HT (b), and NKA (c). Mucosal strips dissected from bladder tissues treated with antibiotic-free RPMI (control, open circles) and UTI89 (2.0 × 108 CFU/ml) (closed circles) showed no significant difference in contractile response to ACh and NKA. However, there was a significant increased contractility to 5-HT in UTI89-treated mucosal strips compared to the control. Data are shown as mean ± SEM from *n* = 7–17 bladders for each group (two-way ANOVA followed by Bonferroni test). *P < 0.05 and **P < 0.01, UTI89 compared to the control.

Mucosal strips isolated from control porcine bladder tissue demonstrated a dose-dependent increase in contractility in response to ACh (Fig. 4a), 5-HT (Fig. 4b), and NKA (Fig. 4c). The maximum responses (E_max_) to 5-HT and NKA in control tissues were ~56% and 82%, respectively, of the maximum response to ACh. Similar contractility was also observed in strips isolated from UTI89-treated bladders for both ACh (Fig. 4a) and NKA (Fig. 4c). Interestingly, there was a significant increase in the contractile response to 5-HT in strips isolated from UPEC treated bladders, in comparison to the control (*P* = 0.012 at 10^−6^ M, *P* = 0.024 at 10^−5^ M, *P* = 0.0021 at 10^−4^ M 5-HT, Fig. 4b). UTI89 pre-treatment did not alter potencies, as the EC_50_ values derived from the concentration response curves of ACh, 5-HT, and NKA in UTI89 groups were similar to those of the corresponding controls (Table 3).

**Table 3.** Contractile responses of porcine bladder mucosal strips in response to ACh, 5-HT and NKA.

	ACh			5-HT			NKA		
Group	EC_50_ (µM) (95% CI)	E_max_ ± SEM (% maximum)	*n*	EC_50_ (µM) (95% CI)	E_max_ ± SEM (% maximum)	*n*	EC_50_ (µM) (95% CI)	E_max_ ± SEM (% maximum)	*n*
Control	17.5 (11.7–26.5)	109 ± 4.98	17	0.17 (0.074–0.46)	55.7 ± 6.62	7	0.033 (0.019–0.057)	81.6 ± 4.02	7
Cranberry	15.1 (8.50–2.79)	115 ± 5.43	14	0.16 (0.072–0.35)	56.7 ± 7.13	9	0.037 (0.015–0.088)	86.5 ± 14.6	12
D-mannose	56.1* (31.3–96.6)	117 ± 4.55	12	0.21 (0.11–0.42)	73.0 ± 11.4	7	0.019 (0.0082–0.042)	72.3 ± 6.44	10
UTI89	28.5 (15.6–52.6)	124 ± 6.71	11	0.13 0.035–0.52	91.3 ± 14.6**	9	0.024 (0.0088–0.067)	85.2 ± 5.30	9
Cranberry + UTI89	27.4 (15.9–48.1)	122 ± 5.18	13	0.18 (0.085–0.39)	87.2 ± 13.5^#^	7	0.029 (0.016–0.055)	79.2 ± 3.93	10
D-mannose + UTI89	19.0 (8.72–57.8)	137 ± 3.79	10	0.12 (0.052–0.26)	86.3 ± 14.1	10	0.013 (0.0044–0.034)	95.6 ± 13.4	10

This table summarizes the EC_50_, EC_50_ (95% CI), E_max_ ± SEM, and *n* values for the mucosal contractility in response to ACh, 5-HT, and NKA in all the treatment groups.

* *P* < 0.05 for D-mannose alone compared to the control.

** *P* < 0.01 for UTI89 compared to the control.

# *P* < 0.05 for cranberry with UTI89 compared to cranberry alone. Data are shown as mean ± SEM from *n* = 7–17 bladders for each group (two-way ANOVA followed by a Bonferroni test).

### Effect of cranberry and d-mannose on UPEC-induced damage in porcine bladder

Cranberry (Howell et al. 2005) and d-mannose (Schaeffer et al. 1984) have been previously shown *in vitro* to prevent UTI89 binding to the urothelium. Therefore, the effect of cranberry and d-mannose pre-treatment on UTI89-induced urothelial damage was examined in this *ex vivo* model. In the initial assessment of mucosal damage, porcine bladders incubated with both cranberry and d-mannose alone preserved the urothelium with mucosal damage scores that were equivalent to those seen in control tissue (Table 2). Bladders incubated with cranberry plus UTI89 or d-mannose plus UTI89 showed similar level of urothelial damage to bladders treated with UTI89 alone (Table 2).

Caspase-3-IR and 8-oxo-dG-IR were also similar in control tissues compared to those treated with cranberry and d-mannose alone (Table 2). Likewise, UTI89 pre-treated with cranberry or d-mannose showed no effect on UTI89-induced cell death or oxidative stress. As shown in Fig. 3b, bladders treated with UTI89 demonstrated reduced UP expression (Table 2, Supplementary Fig. S2), which was not prevented by pre-treatment of UTI89 with cranberry or d-mannose. Additionally, both cranberry and d-mannose alone did not cause any morphological changes, showing results similar to the control tissues (Table 2).

For the functional study, mucosal strips from bladders pretreated with either cranberry or d-mannose alone exhibited similar contractile responses to ACh, 5-HT, and NKA (Table 3) in terms of potencies and maximum responses. However, an exception was observed with d-mannose, which showed a 3.2-fold reduction in potency in ACh-induced contractile responses (EC_50_ 56.1 µM for d-mannose vs. EC_50_ 17.5 µM for control, *P* < 0.05). Cranberry and d-mannose appeared to slightly inhibit the UTI89-induced increase in contractile response to 5-HT, as the maximum responses in the cranberry + UTI89 and d-mannose + UTI89 were 87.17 ± 13.48% (*P* < 0.05) and 86.30 ± 14.13%, respectively, compared to 91.3 ± 14.6% in the UTI-only group (Table 3).

## Discussion

### The impact of UPEC on the *ex-vivo* porcine mucosa

UTIs are among the most common bacterial infections encountered in clinical practice (Stamm and Norrby 2001). UTIs correlate with a decrease in the quality of life in patients, with women being disproportionately affected (Maxwell et al. 2023). There is a plethora of research determining how UPEC establishes an infection in the bladder (Justice et al. 2004, Anderson et al. 2003, Sharma et al. 2021). However, there is limited understanding of how UPEC, the most common UTI-causing agent, affects the physiology of the urothelium. Therefore, this study researched how UPEC impacts the urothelial barrier integrity, viability, permeability, barrier function, and whether it can induce oxidative stress using an *ex-vivo* porcine bladder model.

In this paper, exposure of the porcine bladder mucosa to UPEC resulted in significant disruption and loss of the urothelium. Extensive tissue damage caused by UPEC led to cell death in the urothelium, seen as a reduction in the urothelial cell layers and significant disruption to the urothelial barrier function (Abraham and Miao 2015). This finding was similar to the cell death observed in our previous renal cell culture model, where in the presence of UPEC tumour necrosis factor-alpha (TNF-α), an inflammatory cytokine responsible for activating apoptosis (Idriss and Naismith 2000), elevated the expression of programmed death-ligand 1 (PD-1) protein (Konesan et al. 2023). There are also studies demonstrating that urothelial apoptosis is a key event in the pathogenesis mediated by UPEC (Klumpp et al. 2006, Chen et al. 2003). While cell death was observed in the porcine bladders treated with UPEC, there is no evidence from this study that this occurred via activation of apoptosis. This is supported by previous studies which have suggested that UPEC can mediate cell death via either apoptosis (Chen et al. 2003) or necrosis (Hong et al. 2013). While caspase-3-IR expression was demonstrated within the urothelial layer of the porcine bladder explants, this expression was similar for all treatment groups, including the fresh, non-treated porcine bladders. This may suggest that UPEC-induced cell death is not mediated by apoptosis, but potentially necrosis.

UPEC also induced significant damage to the uroplakin (UP) layer and tight junction (ZO-1) expression in the porcine urothelium. UP are cell membrane proteins, which form urothelial plaques on the surface of each urothelial cell (Lee 2011), and are essential in maintaining an effective urothelial permeability barrier (Višnjar et al. 2017). Tight junctions such as ZO-1 are localized between urothelial cells and function to form a high-resistance barrier (Khandelwal et al. 2009, Acharya et al. 2004). Previous research has shown UPEC to cause significant damage to the urothelial barrier by decreasing the expression of ZO-1 and reducing transepithelial resistance and therefore, increased urothelial permeability (Taidi et al. 2022). Therefore, disruption to both the UP layer and ZO-1 by UPEC would damage the urothelial barrier function.

Similarly, Chuag et al. demonstrated significantly lower expression of another tight junction protein, E-cadherin, with immunofluorescence in human bladder tissue from patients with UTI compared to controls, thus highlighting the barrier dysfunction of the urothelium associated with UTI patients (Chuang and Kuo 2013). In our previous study, we demonstrated that UPEC also caused substantial disruption to the barrier function of the epithelial cell monolayer in a renal cell line, as evidenced by decreased trans-epithelial electrical resistance (Konesan et al. 2023). This suggests that as well as decreasing urothelial cell viability, UPEC can also damage the barrier integrity, leading to increased permeability of the urinary tract lining.

Another way that this study measured the health of the porcine bladder mucosa in the presence of a bacterial infection was through the level of oxidative stress. Pathogenic invasion is known to increase ROS production at the target site of infection (Spooner and Yilmaz 2011). Under normal conditions, ROS production is low and increased ROS production is necessary for providing defence against bacteria (Spooner and Yilmaz 2011). However, increased ROS can lead to extensive tissue damage that needs to be repaired to restore the barrier function of the bladder (Spooner and Yilmaz 2011). Increased urine levels of 8-oxo-dG have been noted in patients with other urological diseases characterized by inflammation including interstitial cystitis/bladder pain syndrome, and overactive bladder (Jiang et al. 2022a,b, Dokumacioglu et al. 2018). Increased ROS has also been associated with UTIs, as the urine of patients suffering from UTI contained malondialdehyde, a byproduct of oxidative stress (Kurutas et al. 2005). However, the level of ROS production has never been measured in a UPEC-infected *ex-vivo* model. Given the known association of ROS with tissue damage, it was rather surprising to observe that no significant difference was observed between control and UPEC-treated porcine bladders in relation to 8-oxo-dG. Interestingly, the fresh pig bladder tissues had significant ROS production, potentially indicating that the tissue was already in a state of oxidative stress following harvesting and transport to the research laboratory. This suggests that this *ex-vivo* porcine bladder may not be an ideal UTI model to measure ROS production.

### Effects of cranberry and d-mannose on UPEC-induced urothelium damage

Patients suffering from a UTI are commonly treated with antibiotics. However, antimicrobial treatment can result in the development of multidrug-resistant strains (Stamm and Norrby 2001). As a result, the ‘golden era’ of antibiotics is waning, and the demand for alternative remedies is therefore increasing. Clinical trials have investigated the effectiveness of a number of non-antibiotic agents, including cranberry (Jepson et al. 2012) and d-mannose (Cooper et al. 2022) for potentially minimizing the usage of antibiotics (Konesan et al. 2022).

Our previous *in vitro* study demonstrated that cranberry, but not d-mannose, provided protection against UPEC-induced disruption to the epithelial integrity and barrier function in MDCK cells (Konesan et al. 2023). Hence, this paper is a further exploration of whether cranberry and d-mannose can produce similar results in a UPEC-infected porcine *ex-vivo* model.

In contrast to our cell culture study (Konesan et al. 2023), the non-antibiotic agents cranberry and d-mannose did not provide a protective effect against UPEC-induced damage to the urothelial integrity and viability in this study. Additionally, treatment of UPEC with cranberry or d-mannose did not provide a protective effect on the urothelial barrier function as visualized by the damaged UP layer and loss of ZO-1 tight junction expression. Hence, pre-treatment of UPEC with cranberry or d-mannose could not preserve the urothelial integrity, viability, and barrier function. Perhaps the discrepancy between this result and our previous finding is due to utilizing a kidney cell line in the previous *in vitro* experiment (Konesan et al. 2023) versus a pig bladder in this paper.

UPEC has different adhesin molecules, such as the type 1 and type P fimbriae. The type P fimbriae has been associated with establishing an infection within the kidney, via the Pap G adhesin (Lane and Mobley 2007). In contrast, the type 1 fimbriae initiate infection by binding to UP expressed on the urothelium, via the FimH adhesin (Wang et al. 2009). Cranberry has various constituents that can potentially provide an anti-adherence effect against UPEC binding to the epithelium. However, the main compound that has been studied in the literature is proanthocyanidin-A (PAC-A) (Pappas and Schaich 2009, Rajbhandari et al. 2011). PAC-A is proposed to bind to the type P fimbriae of UPEC (Howell et al. 2005, Gupta et al. 2012), which is typically associated with UPEC binding in the kidney. Since our previous study utilized a kidney cell line (MDCK) (Melican et al. 2011), this likely explains cranberry’s favourable outcome when challenged with UPEC in that model vs. the results from this porcine bladder model.

In contrast, d-mannose prevents adhesion of the type 1 fimbriae to UP (Bouckaert et al. 2005). Although d-mannose was hypothesized to protect the urothelium against UEPC based on this mechanism of action, this was not observed in the current *ex-vivo* porcine bladder model. Previous studies have demonstrated that synthetic mannosides have much greater affinity for the FimH ligand than d-mannose, with *in-vitro* adhesion of UPEC to the human bladder cell line being inhibited by a 100-fold lower concentration of synthetic mannosides compared to the concentration required for d-mannose (Wellens et al. 2008). Although mannosides have shown a greater pharmacological profile *in-vitro* in comparison to d-mannose alone, no mannosides have entered clinicals trial yet. It is possible that a higher concentration of d-mannose may have been effective in protecting the urothelium from UPEC, but our previous study (Konesan et al. 2023) suggested that higher concentrations of d-mannose were toxic to the urothelial cells.

Excessive tissue damage and loss of the permeability barrier in the bladder tissue can potentially cause functional loss, including the disruption to the contractile functions of the mucosal layer. This disruption may alter mucosal contractility responses from several receptors, including muscarinic (Moro et al. 2011), 5-HT (Moro and Edwards 2016), and tachykinin receptors (Grundy et al. 2018). Subtypes of these receptors, located on the detrusor muscle and responsible for strong muscle contractions, have been identified as the M_3_, 5-HT_2A_ and neurokinin NK_2_ receptors. The potential roles of these receptors in mediating bladder mucosal contraction have garnered more attention in recent years. The structures believed to contribute to mucosal contraction include the muscularis mucosae (Fry and Vahabi 2016) and myofibroblasts (Abbasian et al. 2019). It was hypothesized that UPEC-induced tissue damage would increase smooth muscle and/or myofibroblast excitation, manifested by increased contractility of the mucosal layer in response to excitatory mediators such as ACh, 5-HT, and NKA. Therefore, in this study, bladder mucosal strips were analysed for contractile responses to ACh, 5-HT, and NKA.

Stimulation of muscarinic receptors was previously thought to be mediated exclusively by ACh released from cholinergic nerves in the detrusor muscle (Abrams et al. 2006). However, more evidence suggests that ACh is also released from the urothelium via a non-neuronal mechanism (McLatchie et al. 2014). In the urothelium and lamina propria of human bladders, all muscarinic receptors (M_1_ to M_5_) have been identified at the mRNA level, with particularly high expression of M_2_ and M_3_ receptors (Mansfield et al. 2005, Bschleipfer et al. 2007, Ochodnický et al. 2012, Mukerji et al. 2006, Tyagi et al. 2006). Altered responsiveness to cholinergic stimulation in the bladder with UTIs has been reported. For instance, Werkstrom et al. demonstrated that carbachol produced increased contractile responses in the detrusor layer from children with a history of UTI (Werkstrom et al. 2000). In the current study, only mucosal strips treated with d-mannose alone showed a slight reduction in the potency of ACh-mediated contractile responses. However, there was no significant difference in response to ACh between UPEC and control, nor between cranberry or d-mannose pre-treated with UPEC compared to UPEC alone, indicating that changes in contractile elements and signalling pathways under various treatment conditions are negligible.

5-HT is considered an inflammatory mediator in the periphery and is released from platelets and mast cells after tissue injury (Dray 1995). It exerts a direct effect on sensory C-fibres (Moalem et al. 2005). The presence of multiple subtypes of serotonergic receptors on primary afferent nociceptors in the bladder indicates that serotonin may directly produce pain by activating receptors on Aδ- and C-fibres (Sommer 2004). 5-HT enhances bladder afferent firing and nociceptive processing via the stimulation of 5-HT_3_ receptors (Hall et al. 2015, Konthapakdee et al. 2019), which may be associated with pain in the bladder during UTIs. The urinary bladder expresses a wide range of 5-HT receptors with excitatory and inhibitory effects, depending on receptor subtypes and species. However, the contractile response to serotonin in the bladder is generally accepted to occur via 5-HT_2A_ receptors (Moro and Edwards 2016). In this study, the addition of 5-HT induced a concentration-dependent increase in contractile response for all treatment groups. Interestingly, the UPEC-infected bladder developed a significantly higher contractility response to 5-HT in comparison to the control (for concentrations between 10^−6^ to 10^−4^ M). This was also the case for bladders incubated with cranberry and UPEC, which demonstrated a significantly higher contractile response to 5-HT compared to cranberry alone. Serotonin operates within a complex system with many different excitatory and inhibitory pathways (Deen et al. 2017). It is likely that UPEC caused damage to the inhibitory pathways, leading to the enhancement of the contractility response in the presence of UPEC. It cannot be ruled out the possibility that 5-HT regulates the release of urothelium-derived inhibitory factors, which have been reported to modulate bladder contractile responses (Matsumoto-Miyai et al. 2016). UPEC may have disrupted urothelial cells, affecting the production or release of these inhibitory factors.

Another neurotransmitter explored in this research is NKA, which is known to be a potent contractile agonist via the tachykinin receptors (Regoli et al. 1994). In the human bladder, only the NK_2_ receptor has been observed to mediate contractions, with distribution primarily throughout the detrusor layer, sparsely over blood vessels, but not in the urothelium (Sellers et al. 2006). Most studies on contractility studies examine the role of NKA in the detrusor layer, rather than the urothelium and mucosal layer. However, Sadananda et al. demonstrated that in rodent bladder, mucosal contraction in response to NKA is directly mediated via NK_2_ receptors (Sadananda et al. 2008). Their research highlighted that these contractile elements likely include the α-smooth muscle actin immunoreactive layer below the urothelium, identified as myofibroblasts (Sadananda et al. 2008). Similar to the ACh results, bladder mucosal strips did not exhibit an altered contractile response to NKA caused by UPEC in comparison to the control strips.

One of the main strengths of this study involved using a porcine bladder model, which is similar to the human bladder anatomically, physiologically and histologically (Nielsen et al. 2019). However, a limitation of this model involved the absence of an intact blood supply. The lack of blood supply means that it was not possible to examine the interaction between UPEC treatment and recruitment of inflammatory cells in response to tissue damage. This may also provide an additional reason for minimal to no oedema observed in this model. Perhaps an *in vivo* model could be established to examine the effect of this inflammatory cell recruitment on urothelial tissue damage in a future study. Several murine *in vivo* models have demonstrated this in the literature (Zhao et al. 2009, Frick-Cheng et al. 2020), but it has not yet been shown in the porcine model, which is more homologous to the human bladder.

Another limitation is the lack of information regarding the exact concentrations of cranberry and d-mannose used in this *ex vivo* study, particularly how these correspond to the levels entering the urinary bladder after oral ingestion. Proanthocyanidins (PAC) have been identified as the primary active ingredient responsible for cranberry’s ability to inhibit the adhesion of P-fimbriae from UPEC (Gupta et al. 2007, Konesan et al. 2022, Gupta et al. 2012). Valentova et al. reported low levels of PAC metabolites in urine following the consumption of 1.2 g of dried cranberry juice, with only 0.078% to 5% of these metabolites being excreted in urine (Valentova et al. 2007). These data are insufficient to determine the precise concentration of cranberry that reaches the bladder. As a natural product, cranberry has undergone less pharmacological characterization compared to conventional drugs, underscoring the need for further research to determine its pharmacokinetics.

d-mannose has a structure similar to the typical bacterial binding sites on bladder uroplakins. Therefore, once present in urine at sufficiently high concentrations, d-mannose can interact with mannose-sensitive components of UPEC, potentially mitigating the bacterium’s pathogenic effects (Konesan et al. 2022). d-mannose has been shown to enter the urine from the bloodstream within 60 min (Ganda et al. 1979, Wood and Cahill 1963). For example, Alton et al. administered a dose of 0.1 g of d-mannose per kg of body weight to female rats, with the compound excreted into the urine within 30–60 min (Alton et al. 1998). However, these findings may not accurately reflect the concentration of d-mannose in the bladder, and direct comparison with the concentrations used in our study is not feasible.

Taken together, our results demonstrate that UPEC was able to disrupt the urothelial integrity, barrier function and permeability of the porcine bladder. This evidence that UPEC can cause significant damage to the mucosa may support the hypothesis that urothelial damage is the trigger for UTI symptoms of pain, frequency, and urgency as a result of bladder afferent nerve activity. In this study, neither of the two non-antibiotic agents, cranberry nor D-mannose, was able to provide a protective effect in preserving the urothelial integrity in the porcine bladder. These findings suggest that perhaps these agents cannot block the binding of UPEC to the urothelial surface in the porcine bladder model, consequently failing to prevent bacterial-induced damage to the urothelial barrier.

## Supplementary Material

ftae026_Supplemental_File

## Data Availability

The authors confirm that the data supporting the findings of this study are available within the article and its supplementary materials.
