# Supplementary material for: Uropathogenic Escherichia coli causes significant urothelial damage in an ex vivo porcine bladder model, with no protective effect observed from cranberry or d-mannose
Source: Pathog Dis. 2024 Oct 3;82:ftae026. doi: 10.1093/femspd/ftae026 (PMC11879300; doi:10.1093/femspd/ftae026)
Supplement: ftae026_Supplemental_File [file ftae026_supplemental_file.pdf]

**Supplementary Table I**: Primary and secondary antibodies used for DAB immunohistochemistry staining and immunofluorescence.

| Antibody | Target | Antibody description | Dilution | Manufacturer and catalogue No |
|---|---|---|---|---|
| **Primary** | UP (IF) | Rabbit anti-UP III | 1 in 100 | Abcam ab187646 |
| | ZO-1 | Rabbit anti-ZO-1 | 1 in 100 | Invitrogen 61-7300 |
| | 8-oxo-dG | Mouse anti-DNA/RNA damage | 1in 1000 | Abcam ab62623 |
| | Caspase-3 | Rabbit anti-cleaved caspase 3 | 1 in 500 | Abcam ab32042 |
| **Secondary** | UP (IF) | Anti-Rabbit IgG H&L (Alexa Fluor® 488) | 1 in 200 | Abcam ab150129 |
| | ZO-1 (IF) | Anti-Rabbit IgG H&L (Alexa Fluor® 594) | 1 in 200 | Abcam ab150080 |
| | 8-oxo-dG | Goat anti-mouse IgG | 1 in 200 | Abcam 6708 |
| | Caspase-3 | Donkey anti-Rabbit IgG | 1 in 200 | Abcam 6701 |

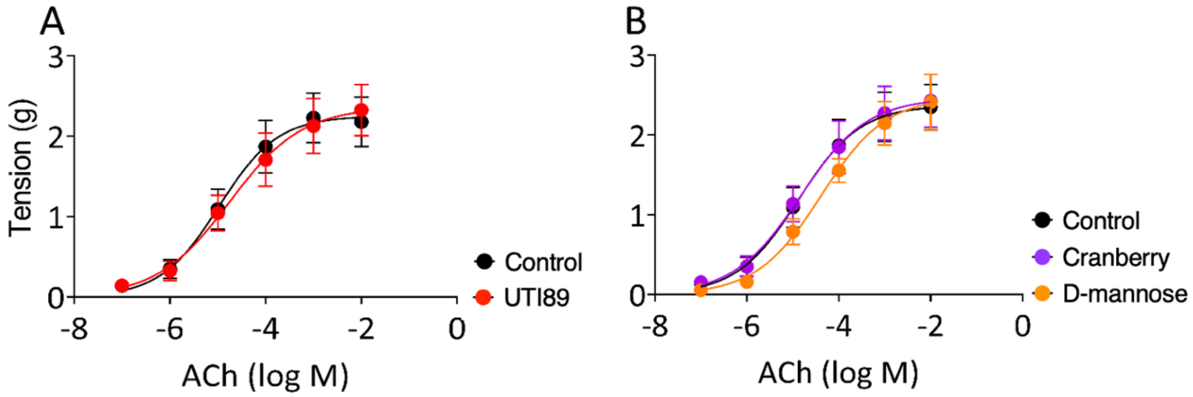


**Supplementary Figure 1**. **Concentration-response curves of porcine bladder mucosal strips to ACh.** Contractility was measured and expressed as gram tension. The curves generated from bladder tissues pre-treated with UTI89 (**A**) or cranberry (**B**) were superimposed with those of the control tissues. Although the curve from bladder tissues pre-treated with D-mannose showed a slight rightward shift in the ACh concentration-response curve, the maximum response at ACh $10^{-2}$ M was nearly identical to that of the control tissues (**B**), similar to the UTI89 and cranberry groups. Therefore, the contractile tension induced by ACh $10^{-2}$ M in individual mucosal strips was taken as the maximum response.

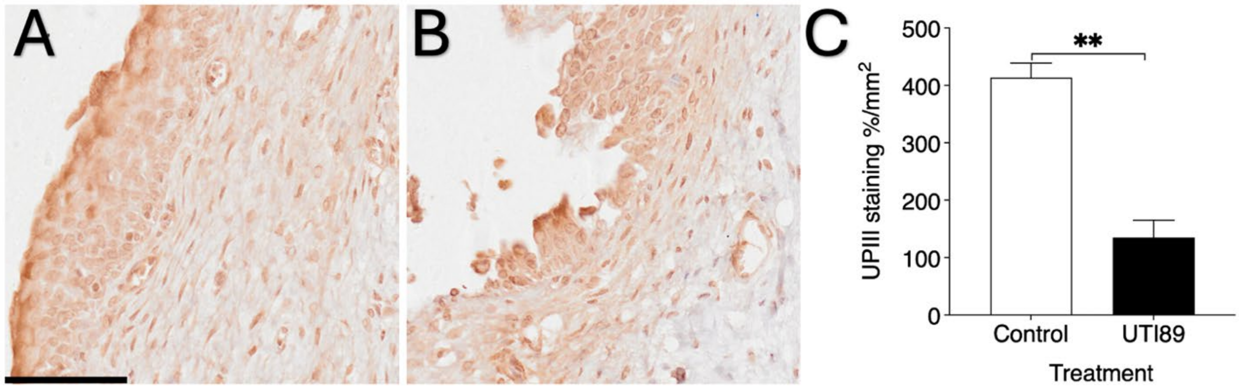


**Supplementary Figure 2. Severity of damage to the uroplakin layer induced by UPEC UTI89**. DAB staining was used to visualise the uroplakin (UP) layer with control (antibiotic-free RPMI) (**A**) or with UTI89 ($2.0 \times 10^8$ CFU/mL) (**B**). Panel (**C**) presents the severity scores of UP layer damage comparing the control and UTI89-treated groups. Data were collected from n = 5 individual experiments. ***p* < 0.01, UTI89 compared to the control (unpaired t-test). The scale bar represents 100 µm.
